# Phototransformation of Amlodipine: Degradation Kinetics and Identification of Its Photoproducts

**DOI:** 10.1371/journal.pone.0109206

**Published:** 2014-10-03

**Authors:** Anna Jakimska, Magdalena Śliwka-Kaszyńska, Piotr Nagórski, Jacek Namieśnik, Agata Kot-Wasik

**Affiliations:** 1 Department of Analytical Chemistry, Faculty of Chemistry, Gdańsk University of Technology, Gdańsk, Poland; 2 Department of Organic Chemistry, Faculty of Chemistry, Gdańsk University of Technology, Gdańsk, Poland; Osaka University Graduate School of Medicine, Japan

## Abstract

Nowadays, monitoring focuses on the primary compounds and does not include degradation products formed during various biological and chemical processes. Transformation products may have the same effects to human health and the environment or sometimes they can be more toxic than the parent compound. Unfortunately, knowledge about the formation of degradation products is still limited, however, can be very important for the environmental risk assessment. Firstly, the photodegradation kinetic of amlodipine was investigated in two experimental conditions: during the exposure to solar radiation and during the exposure to the light emitted by the xenon lamp. In all cases degradation of amlodipine followed a pseudo-first-order kinetics. In the next step, identification of transformation products of amlodipine formed during the exposure to xenon lamp irradiation was performed using ultra high performance liquid chromatography quadrupole time-of-flight mass spectrometry (UHPLC-QTOF-MS). As a result sixteen photoproducts were identified, their structures were elucidated and ultimately the transformation pathway was proposed. Fifteen compounds (out of 16 photoproducts) were newly identified and reported here for the first time; some of those compounds were formed from the first photoproduct, amlodipine pyridine derivative. Several analytes were formed only in acidic or basic conditions. Furthermore, the occurrence of amlodipine and its identified degradation products was investigated in environmental waters. Only one out of 16 compounds was found in wastewater effluent. The possibility of the sorption of examined analytes to sewage sludge particles was discussed based on QSAR.

## Introduction

There are many research which focus on the presence of pharmaceuticals in the aquatic environment and the removal efficiency of these emerging contaminants during wastewater processes in wastewater treatment plants (WWTP) is limited [1]. However, the lack of the parent compound in the effluent is not equal to its total elimination and/or lack of transformation products (TPs). During the wastewater treatment, or later in the environment itself various stable TPs may be formed under the influence of different factors and reactions. Therefore, it is necessary to consider both parent compounds and products of their transformation which appear in the aquatic environment.

The result of the chronic exposure to low doses of pharmaceuticals and their TPs still remain unknown. Their level in the environment is not regulated, and thus these compounds are considered environmentally hazardous [2]. It was found that some drugs (e.g. ibuprofen) exhibited toxicity in relation to living organisms. Furthermore, it was proved that some of the formed TPs were toxic as well [3], [4]. Environmental degradation of xenobiotics depends on their structure as well as weather or natural conditions (including sunlight, temperature or microorganisms). The degradation process substantially reduces the potential of a drug to induce a pharmacological effect, however, TPs may in some cases exhibit similar activity and be more stable than the parent compounds [5]. The stability of the compounds in the aquatic system plays a major role in determining their potential to cause negative effects in the environment. There is a gap in the knowledge of TPs of various drugs and their impact on the environment and organisms as well [6]. Consequently, there is a clear need to investigate the environmental fate of different pharmaceuticals.

The compound selected for degradation study was amlodipine - a long-acting calcium channel blocker used to lower blood pressure and to treat anginal chest pain [7]. It is one of the most often prescribed drugs popularly known as Norvasc [8], and is used in minimum dose of 5 or 10 mg per day depending from the therapy (mono- or combined therapy) [9]. It was observed that among people between 35–64 years about 30% of the US population and 44% of the European population suffer from hypertension [10]. Additionally, it was estimated that around 1 billion people worldwide had hypertension by 2000, and it is expected that this number would increase to 1.5 billion people by 2025 [11], [12]. Even though, amlodipine undergoes oxidative metabolism [9], it is partially excreted with urine in unchanged (∼6%) form [13] and can be found in e.g. hospital effluents or wastewaters in up to tens of ng L^−1^ [14]. Furthermore, the published research showed that amlodipine causes toxic effect such as inhibition of the regeneration of a hypostome, tentacles and foot in *Hydra vulgaris* at the exposure level of 10 µg L^−1^ [15]. The other study revealed that the main photoproduct of amlodipine, its pyridine derivative, exhibited a stronger toxic potential than the parent drug in *Ceriodaphnia dubia* [16]. However, there is very little information in the literature on amlodipine and its transformation products. The compound formed by the aromatization of the ring (pyridine derivative) is the only photoproducts known and described in the literature so far [16], [17], [18]. A few studies were performed for thermal degradation of amlodipine showing the formation of 3 [19] or 5 main transformation products [20].

The most important processes which may occur in the aquatic environment include biodegradation, photodegradation and hydrolysis. Biological processes allow only a limited transformation of pharmaceuticals due to their biopersistance [21]. Biochemical reactions can occur only in a certain way, whereas photochemical processes are less predictable, particularly when radicals are involved, which do not occur in the case of biochemical processes [21], [22]. Therefore, it can be expected that in the aquatic environment photodegradation will be more important mechanism of pharmaceuticals transformation [23], [24], especially in case of compounds which structure contains aromatic rings, heteroatoms and other functional groups that can absorb the sunlight (direct photolysis) or react with active molecules •OH, ^1^O_2_, ROO•, ^3^DOM*, e^−^_aq_ generated by photosensitizers (indirect photolysis) [25], [26].

Nowadays, investigation of suspected compounds e.g. TPs, their structure elucidation and/or confirmation requires application of highly accurate, selective and reliable techniques. To meet the present expectations, the hyphenated techniques based on liquid chromatography and high resolution mass spectrometry (LC-HRMS) have become a powerful tools [27]. Such instruments as LC- quadrupole time-of-flight mass spectrometry (LC-QTOF-MS) or LC-Orbitrap-MS provide accurate mass measurements, information on isotope pattern of a compound due to their high resolution and full scan product ion spectra, and thus provide structural information of the analyte [27], [28].

In light of these concerns and the lack of scientific literature on phototransformation of a widely prescribed drug, amlodipine, the main aim of this study was to identify transfromation products formed during the continuous exposure of amlodipine water solution to UV/VIS irradiation emitted by xenon lamp. Furthermore, studies on photodegradation kinetics with the simulation of the conditions in natural aqueous waters were undertaken. The final analysis were performed with the application of LC-QTOF-MS technique providing reliable results with mass error <5 ppm. To the best of our knowledge, this is the most extensive study on photodegradation products of amlodipine including kinetics and structure elucidation followed by the proposal of its phototransformation pathway. As a result 16 degradation products were identified. The first product identified was pyridine derivative of amlodipine (known in the literature), and the other fifteen were compounds formed from either the parent molecule or its first degradation product and were reported here for the first time. The proposed structures of newly identified compound were elucidated and verified on the basis of data obtained during LC-QTOF-MS analysis including fragmentation mass spectra of (pseudo-)molecular ions, accurate mass measurements and isotope patterns of each compound. Finally, the presence of amlodipine and its transformation products was investigated in various environmental waters. The application of QSAR allowed the estimation of possible sorption of target compounds on sewage sludge particles.

## Materials and Methods

### Reagents

Analytical standards of amlodipine (AML) were purchased from Sigma-Aldrich (Poznań, Poland). LC-MS grade methanol (MeOH) was obtained from Sigma-Aldrich (Poznań, Poland). Formic acid was from Merck (Warsaw, Poland). Ultrapure water was obtained from a HLP5 system (Hydrolab, Poland). MeOH used for extraction were LC grade and were purchased from Merck (Warsaw, Poland). Individual stock solution of AML at concentration level of 1 g L^−1^ was prepared in methanol and was stored at −80°C.

### The irradiation experiments

#### Identification of TPs of AML

Degradation experiments were performed using a small-scale system that consisted of a cylindrical photoreactor (25 mL) equipped with quartz window and cooling system, external light system and optical filters. The radiation was emitted by 1000W xenon lamp (627H, Oriel) which transmitted light of wavelength ranged from 250 to 1000 nm. Optical filters (UG1, Schott AG) were used for cutting off the IR irradiation. This equipment was previously applied for identification of phototransformation products of three other pharmaceuticals (ketoprofen, ibuprofen and furosemide) in river water samples [29] and by Górska et al. [30] for photodegradation of phenol.

The temperature of the samples during the experiment was maintained at 10°C. The photodegradation experiment was performed in river water (free from the targets) at three different pH (3, 10 and no pH adjustment). The concentration of AML was 1 mg L^−1^ since higher initial concentration provides TPs in higher amount (this increases the possibility of proper identification and structure elucidation of TPs). 25 mL of aqueous sample containing AML was stirred magnetically during the irradiation experiment. Aliquots of about 500 µL were collected during the experiment every 15 minutes, filtered through a 0.45 µm nylon syringe filter (Rockwood, USA) and directly subjected to UHPLC-QTOF-MS analysis for further data evaluation.

#### Degradation kinetic studies

Photodegradation kinetics were designed in two ways: (1) as a result of an exposure to xenon lamp light (in order to ensure the independence of the degradation process from the weather conditions that may affect the experiment; xenon lamp emits condensed radiation, which range overlaps with the range of solar radiation), (2) as a result of a continuous exposure to solar radiation (in order to simulate conditions similar to those in the natural environment).

Degradation under continuous exposure to solar radiation was performed for samples characterized by eight different matrices: wastewater influent and effluent, untreated and treated water, river water, methanol, ultrapure water for pH 3 and pH 10. For this purpose, specific matrix was spiked with AML at the concentration level of 1 mg L^−1^. The samples were kept in glass vials (25 mL) exposed to natural sunlight; the experiment was performed during the summer 2013 in order to provide sufficient sunlight intensity. The aim was to investigate the influence of the matrix and pH on degradation rate of AML.

Degradation under xenon lamp irradiation was performed only for river water samples (as well as for the identification of TPs). To evaluate the degree of degradation of AML, UPLC-QTOF-MS analysis was performed.

### UHPLC-QTOF-MS analysis

Both chromatographic conditions and mass spectrometer parameters were previously described in other publication [29]. Briefly, analysis was performed on Agilent 1290 UHPLC system with XTerra MS C18 Column (30 mm×2.1 mm; 3.5 µm). The mobile phase A was ultrapure water with 0.05% FA and B was MeOH at a flow rate 0.4 ml min^−1^ in gradient elution (95% A to 0% A in 15 min, back to 95% A in 1 min and kept at 95% A for 5 min). The column was maintained at 22°C and the injection volume was 5 µL.

The UHPLC system was coupled to a hybrid quadrupole time-of-flight (QTOF) mass spectrometer (Agilent 6540 Series Accurate Mass QTOF-MS) with Dual ESI interface and operated in positive ion mode. Operation conditions were as follow: sheath gas temperature 400°C at flow rate of 12 L min^−1^, capillary voltage 4000 V, nebulizer pressure 20 psig, drying gas 10 L min^−1^, gas temperature 325°C, skimmer voltage 45 V, octopole RF peak 750 V and fragmentor voltage 100 V. Analysis were performed using MS/MS or TargetMS/MS mode with variable collision energies (10, 20 or 30 V; however, 20 V was the optimal value for all the target compounds) and in mass range 50–1000 m/z. The instrument was operated in the 4 GHz high-resolution mode and acquisition rate was 1.5 spectra per second. Acquisition data were processed with Agilent MassHunter Workstation software.

### Sample collection and preparation

Aqueous samples collected from the Gdańsk area were: influent and effluent water samples from a wastewater treatment plant (WWTP), and river water from Kacza River exposed to anthropogenic activity. Samples were collected in pre-rinsed amber glass bottles, filled to the brim in order to reduce analytes transition to the gas phase. They were stored at 4°C and analyzed within 24 h for the presence of AML and identified photoproducts.

Samples were prepared by solid phase extraction (SPE) with the application of Oasis HLB cartridges (500 mg, 6 mL; Waters). SPE cartridges were preconditioned with 5 mL of MeOH and 5 mL of ultrapure water. 1 mL 0.1 M EDTA/100 mL sample was added before loading. A volume of 100 mL, 200 mL and 500 mL of influent, effluent and river water, respectively, were loaded on the sorbent at a flow rate of 5 mL min^−1^ (if the sample contained solid particles, it was first filtrated by a cellulose filter). Later, the sorbent was dried for 15 min. The analytes retained on sorbent were eluted with 6 mL (3×2 mL) of MeOH and obtained extracts were evaporated to dryness in a stream of N_2_. Dry residues were redissolved in 1 mL of MeOH/H_2_O (10∶90, v/v) and 5 µL of the final extract was injected into the UHPLC-QTOF-MS system.

## Results and Discussion

### Photodegradation kinetics of AML

**Figure 1:**
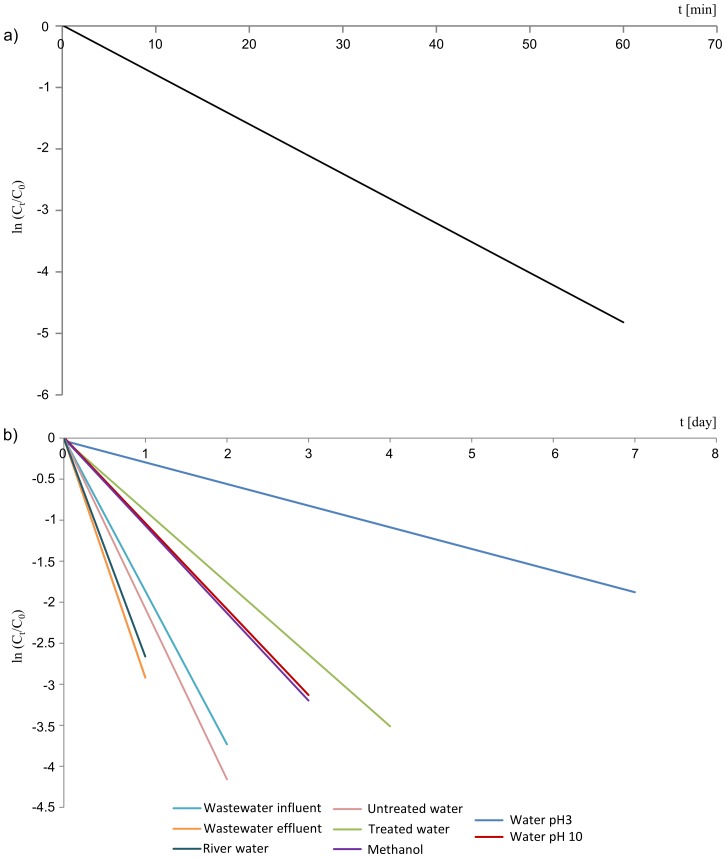
Kinetic curves for AML obtained for various conditions: exposure to xenon lamp irradiation (a) and exposure to natural sunlight including eight different matrices (b).

The reaction kinetics included the calculation of the degradation rate and the assessment of the influence of external factors on this parameter. This was determined based on the dependence of the product formation/substrate decrease rate on initial concentration of the substrate and the obtained relation allowed determining the reaction kinetic equation. The degradation reaction kinetics, i.e. the reaction order, the rate constant and the half-life were determined for AML for the degradation process induced by xenon lamp irradiation, as well as the degradation process caused by continuous exposure to sunlight, for samples characterized by various matrices. All experiments lasted until the decrease of AML concentration at least below 10% of the initial value. The results are presented in Table 1 and in Figure 1a.

**Table 1:** Photodegradation rate constants (*k*) and half-lives (t_1/2_) of AML in various matrices exposed to solar or xenon lamp irradiation.

Matrix	Solar irradiation	
	*k* (day^−1^)	*t_1/2_* (day)
Wastewater influent	1.87	0.4
Wastewater effluent	2.92	0.2
River water	2.66	0.3
Untreated water	1.79	0.4
Treated water	0.87	0.8
Methanol	0.55	1.3
Ultrapure water pH 3	0.32	2.2
Ultrapure water pH 10	0.96	0.7
	**Xenon lamp irradiation**	
	***k* (min^−1^)**	***t_1/2_* (min)**
River water	0.08	8.8

The photodegradation of AML in river water induced by the irradiation with xenon lamp followed a pseudo-first-order kinetic (Table S1), which was determined by regression analysis, and the time-based pseudo-first-order rate constant (*k*) was determined according to the Eq. (1):
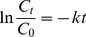
(1)where *C_t_* is the concentration of the compound at the irradiation time t and *C_0_* is the initial concentration of the analyte.

Half-life *t_1/2_* was calculated using Eq. (2) which was derived by modifying Eq. (1) and replacing *C_t_* with *C_0_/2*:
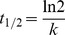
(2)


Even though the signal from AML was not observed after 60 min of the exposure to xenon lamp light (*t_1/2_* = 8.8 min), the experiment was performed for additional 30 min (total irradiation time was 90 min) in order to degrade the pyridine derivative of AML, which was created after 15 min of the exposure, and to identify additional transformation products. The obtained results are consistent with the literature since the photodegradation reactions induced by a constant irradiation are usually followed by the first-order kinetics [31].

The photodegradation of AML in various matrices induced by the solar radiation followed pseudo-first-order kinetics as well (Table S1). The parameters *k* and *t_1/2_* were determined analogously to the xenon lamp experiment and the results are presented in Table 1 and in Figure 1b. The degradation rate depended not only on the structure of the compound, the type and amount of functional groups, but also on the matrix. A significant increase of degradation rate was observed for samples with aqueous matrices compared to methanol. This is probably associated with the radical reactions which occur in aqueous medium (as a result of exposure to radiation in aqueous samples reactive hydroxyl radicals are formed and react with the molecule easily triggering the degradation of the compound). Decreasing the pH of the water sample (acidic conditions) inhibited the degradation process since it is a known method of inhibiting microbiological activity, which limits the decomposition of compounds.

The first signal that suggested the occurrence of the degradation process was a decrease in the AML concentration. However, it cannot be unequivocally stated that the observed changes in the amount of the compound in the water samples were a result of its strict degradation. Therefore, in order to confirm the degradation of AML, the decrease of the AML concentration followed by the identification of products formed during the irradiation experiment was observed with the application of UHPLC-QTOF-MS.

### Identification of photoproducts of AML

The identification experiment was performed under xenon lamp irradiation providing independence of the formation of degradation products from external conditions (e.g. variations of temperature or light intensity) which may adversely affect the identification procedure. Not every wavelength reaches the Earth at the same intensity and the contribution of the low wavelengths (below ∼250 nm) is limited and dependent inter alia on the latitude. In the aquatic environment direct and indirect photolysis occurs simultaneously. The occurrence of direct photolysis depends on the overlap of the absorption spectrum of the compound with the wavelength range emitted by the radiation source, and therefore, compounds with the absorption maxima in the range of low wavelengths will more probably undergo to indirect photolysis. However, in the case of a xenon lamp irradiation the occurrence of direct photolysis is equally possible because it emits radiation of constant intensity. Amlodipine has two absorption maxima: 240 and 360 nm (Figure S1) and even that only the second one overlap the xenon lamp wavelength range (∼250–1000 nm) it was considered sufficient to degrade the analyte.

The identification of photoproducts was performed only in river water samples exposed to xenon lamp radiation. This matrix was chosen for several reasons: river water is representative for the aquatic environment, it is a source of drinking water, a place of wastewater discharges and popular recreational place for people. Identification of photoproducts was performed by UHPLC-QTOF-MS analysis of river water sample spiked with AML exposed to a xenon lamp radiation and collected at specified intervals. Firstly, (pseudo-)molecular ions in the full scan acquisition mode which occurred during the experiment and could respond to probable photodegradation products were selected. Later, the analysis in TargetMS/MS mode was performed in order to obtain the fragmentation spectra of selected compounds. As a result, it was possible to verify the pre-selected compounds and elucidate the structures of photodegradation products. In order to provide reliable results acceptable accuracy mass measurement error was below 5 ppm for (pseudo-)molecular ions and 10 ppm for fragment ions. This analytical approach, including specific identification evidence (e.g. MS, MS/MS, reference standard, experimental data, etc.) as indicated by other authors [32], [33], is a safe way to very accurate identifications and helpful in elucidating the structure of ‘predicted’ compounds, in particular when the analytical standard is not available and the analyte is not present in available databases [33].

**Figure 2:**
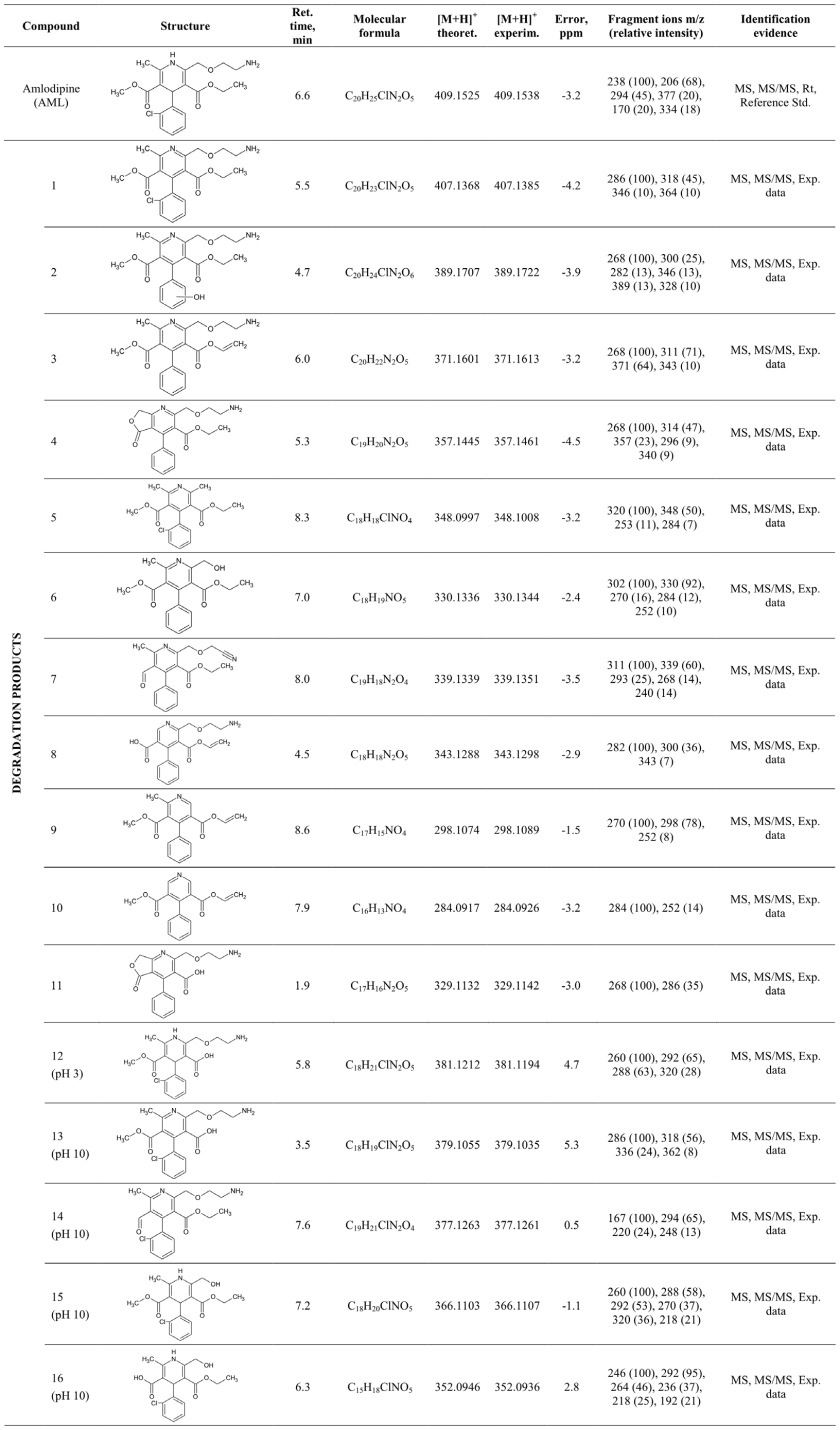
Degradation products of amlodipine identified during the irradiation of river water samples by xenon lamp light.

Xenon lamp irradiation of the studied AML resulted in the formation of new chromatographic peaks corresponding to transformation products generated during the photolysis process. Xenon lamp irradiation of AML produced a further sixteen new products (AML 1–16), and fifteen of them are reported here for the first time. All photoproducts had lower molecular weights than the parent compound and photoinduced dimerization was not observed. Detailed results presenting the identified compounds are listed in Figure 2. The experimental masses (m/z) deviation from the theoretical molecular masses for protonated [M+H]^+^ transformation compounds ranged from −4.5 to 5.3 ppm. The accurate mass MS/MS spectra for identified photoproducts were obtained by fragmenting the selected protonated molecule [M+H]^+^ at specific m/z value. Based on the fragmentation patterns observed in the MS/MS spectra the molecular formula and structure of the products were then tentatively assigned. These accurate mass measurements of both precursor and product ions together with the fragmentation pattern of each photoproducts obtained by UHPLC-QTOF-MS analysis were of high importance while verifying the structures and elucidating the transformation pathway of amlodipine transformation. In the main article only two exemplary MS/MS spectra (for AML 1 and 2) are presented; the other spectra (for AML 3 – AML 16) are presented in supplementary materials (Figure S2).

**Figure 3:**
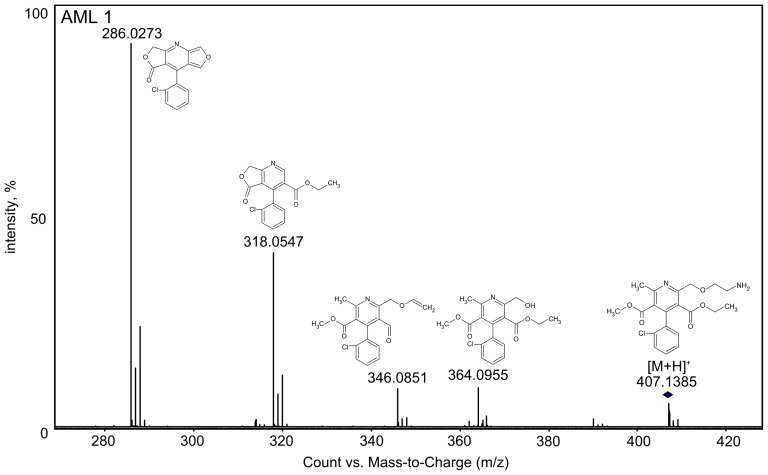
MS/MS spectrum of AML 1 with proposed structures of each fragment ion.

The first photoproduct found was amlodipine pyridine derivative (AML 1, m/z 407.1385). Formation of this product was explained on the basis of a radical cation intermediate according to Fasani's investigation [17]. The structure of this compound was confirmed by the fragment ions obtained during the MS/MS fragmentation (Figure 3). Aromatization of the dihydropiridine moiety is the first step of the amlodipine degradation and this product seems to be the precursor of all further transformations. This photodegradation product has been already identified in the literature [16].

**Figure 4:**
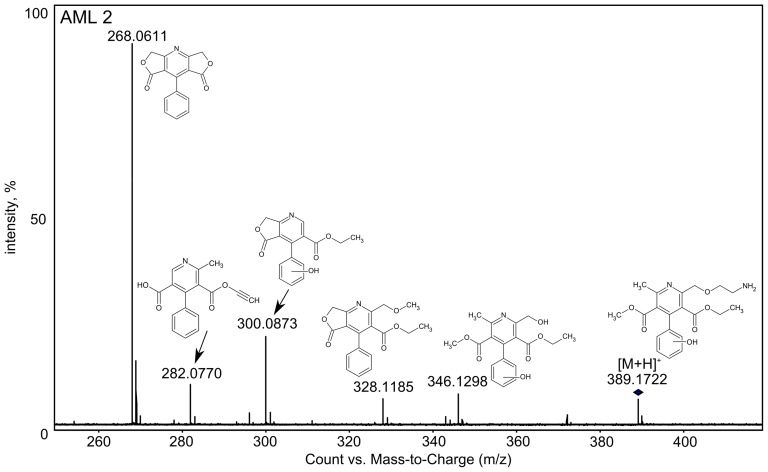
MS/MS spectrum of AML 2 with proposed structures of each fragment ion.

Xenon lamp irradiation of AML solution caused after 15 minutes formation of further seven new products. Formation of AML 2 (m/z 389.1722) is postulated to occur from AML 1 by loss of chlorine atom and hydroxylation of benzene ring by hydroxyl radicals present in the aqueous environment under photolysis [34], and its MS/MS spectrum together with the structures of each fragment ion are presented in Figure 4. In the mass spectrum of product AML 3, the most important fragment is the m/z 371.1613, which corresponds to the elimination of the chlorine atom and loss of molecular hydrogen from the AML 1. The structure of m/z 357.1461 (AML 4) may corresponds to the aromatized amlodipine molecule after elimination of chlorine atom and methyl group resulting formation of lactone fragment.

Another photoproduct detected after 15 minutes of the exposure of water solution to xenon lamp is AML 5. This product with the m/z value 348.1008 may correspond to the derivative after loss of 2-aminoethenol fragment from AML 1 compound. The most important fragment in the mass spectrum of product AML 6 is m/z 330.1344 and may correspond to the disconnection of chlorine atom and etheneamine from the AML 1 product. Compound AML 7 with the m/z value 339.1351 was probably formed by dehalogenation, oxidation and elimination of formaldehyde molecule from AML 1 product, whereas AML 8 (m/z 343.1298) results by loss of methyl substituent and transformation of the methyl ester to carboxylic acid derivative, followed by dehydrogenation.

Compound AML 9, which structure corresponds to the AML 3 byproduct after elimination of the furanylmethyl group (m/z 298.1074), was identified in the sample taken after 30 minutes of continuous exposure of the amlodipine solution. Further irradiation of amlodipine (45 min) revealed formation of another degradation product (AML 10, m/z value 284.0917). This compound was assumed to be the result of elimination of methyl substituent from AML 9 photoproduct.

In the mass spectrum of product AML 11, the most important fragment is the m/z 329.1142, which may corresponds to the hydrolysis of ethyl ester of AML 4 photoproduct.

The irradiation of a sample containing amlodipine in a water solution acidified to the pH 3 led to the formation of another compound AML 12. This product with the m/z value 381.1212 may correspond to amlodipine derivative after hydrolysis of ethyl ester.

Exposure of this drug solution to xenon lamp light in the water at pH 10 caused formation of four photoproducts. Compound AML 13 and AML 14 originated from amlodipine after aromatization (AML 1); however, AML 15 and AML 16 were formed directly from amlodipine. The most important fragment in the mass spectrum of product AML 13 is the ion of m/z 379.1035, which corresponds to hydrolysis of ethyl ester from the parent molecule. The structure of m/z value 37.1261 (AML 14) may correspond to AML 1 product after transformation of the methyl ester group to aldehyde moiety. In the mass spectrum of product AML 15, the most important fragment is the m/z 366.1107, which may correspond to the elimination of etheneamine fragment from the AML. The final compound detected in this solution (AML 16, m/z value 352.0936) was probably formed by hydrolysis of methyl ester from the AML 15 molecule.

Irradiation of amlodipine solution by the xenon lamp has been terminating after 90 minutes. No signals originate from AML was recorded, whereas the presence of several degradation products were still detected in the examined solution.

**Figure 5:**
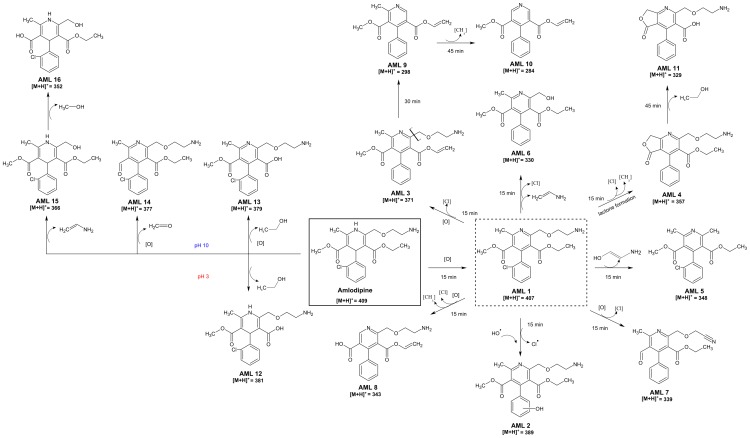
Proposed photodegradation pathway of amlodipine.

On the basis of the degradation products identified in this experiment the hypothetical amlodipine degradation pathway was suggested, and schematically shown in Figure 5.

### The occurrence of AML and its photoproducts in environmental waters

The presence of amlodipine and identified compounds of its phototransformation was investigated in wastewater (influent and effluent) and river water in order to estimate the actual pollution of the environment. The analysis was performed using UHPLC-QTOF-MS system in TargetMS/MS mode. Confirmation of the presence of degradation products was done by using information obtained during the analysis and MS/MS fragmentation of (pseudo-)molecular ion (compliance of retention time (deviation <2%), mass accuracy of (pseudo-)molecular ion (<5 ppm) and minimum two fragment ions (<10 ppm), and relative intensity of fragment ions (deviation <5%)).

Photodegradation products of amlodipine were not detected in the tested samples, except for AML 2 which was present in the wastewater effluent (average peak area ± SD (n = 4)  = 1,1E+05±2,0E+04). These results can be interpreted in various ways. Amlodipine as a parent compound is rarely detected in surface water and wastewater samples [35], [36]; however, it was detected in hospital effluents and in wastewaters from WWTPs at an average concentration level ranged from 36.5 to 93.9 ng L^−1^ [14]. Although, amlodipine is a common drug for lowering blood pressure, it is metabolized [9] and excreted in unchanged form at very low level. This might be the main reason of the non-detection of amlodipine as parent compound in tested samples. Even when AML is present in very small quantities its non-detection may be related to the insufficient sensitivity of applied techniques.

However, there is a possibility that both parent compound and TPs are partially adsorbed on the sewage sludge particles, which are known to sorb certain organic compounds on their surface [37]. The physical-chemical properties of AML and its TPs were calculated using quantitative structure-activity relationships (QSAR) (EPI Suite) [38], and the results are presented in Table 2. It can be noticed that all values of octanol/water partition coefficient, log K_ow_, are lower than 4, which indicate high solubility in water of most of the examined compounds (except for AML 5 which log K_ow_ was 4.47). The calculations of soil adsorption coefficient, log K_oc_, gave more specific results suggesting low or negligible sorption to sewage sludge and moderate or rapid migration to ground water for most of the compounds. The exception was observed for AML 5 (log K_oc_ = 3.44) indicating its moderate sorption do sludge particles, and it was also observed in STP model, which estimated its removal by sewage sludge on a level of 54%. An increased sorption was noticed for AML 15 and AML 16 as well, however, it was considered as insufficient to conclude that the sorption process was the most dominant one in their removal.

**Table 2:** Various physical-chemical properties of AML and its photodegradation products, and their estimated removal with sewage sludge based on QSAR (EPI Suite).

Compound	log K_ow_ (estimated)^a^	log K_oc_ b	Removal by sludge (STP model), %
AML	2.07	2.30	5.57
AML 1	2.72	2.36	3.8
AML 2	1.60	1.91	1.91
AML 3	1.94	1.93	2.10
AML 4	0.42	1.09	1.77
AML 5	4.47	3.44	53.93
AML 6	2.36	1.86	2.65
AML 7	1.55	2.00	1.89
AML 8	−0.07	0.82	1.76
AML 9	2.16	2.17	2.32
AML 10	1.62	1.87	1.92
AML 11	−1.13	−0.47	1.76
AML 12	−0.41	−0.29	1.76
AML 13	1.18	0.81	1.82
AML 14	2.61	2.14	3.35
AML 15	3.53	2.29	13.55
AML 16	3.24	1.43	8.19

a Octanol-water partition coefficient (criteria: log K_ow_ <1 highly soluble in water (hydrophilic);>4 not very soluble in water (hydrophobic)).

b Soil organic carbon-water partitioning coefficient (criteria: log K_oc_ <1.5 negligible sorption to sludge, rapid migration to ground water; 1.5–2.4 low sorption to sludge, moderate migration to ground water; 2.5–3.4 moderate sorption to sludge, slow migration to ground water; 3.5–4.4 strong sorption to sludge, negligible to slow migration to ground water).

Although, the predicted removal of AML and its photoproducts through the sorption to sewage sludge particles was estimated on the level below 6% for most of the analytes, the presence of AML in the sludge was reported previously [36]. In order to determine the actual degree of contamination of the environment it would be valuable to perform further research on the presence of the parent compound together with its TPs on various stages of wastewater treatment and in ground water, where many organic contaminants may migrate resulting in its contamination.

The occurrence of only the photoproduct AML 2 may suggest that the amlodipine was present in the wastewater; however, it degraded or migrated to other parts of the environment e.g. ground water. Ongoing research confirms that tracking the fate of not only amlodipine but also other pharmaceuticals in the aquatic environment wider the knowledge of the environmental pollution.

## Conclusion

The photodegradation of amlodipine was investigated using UHPLC-QTOF-MS. The degradation kinetics of AML was determined for two types of experiments: with the exposure of the samples to solar radiation, and with the application of xenon lamp radiation. It was observed that all the reactions follow a pseudo-first-order kinetics, and the time-based pseudo-first-order rate constants (*k*) and half-lives (*t_1/2_*) were determined for both experiments. The identification of degradation products was the main aim of this research, and it was performed with the application of xenon lamp irradiation in order to independent the experiment from the external conditions, which may influence the experiment, and to provide a constant light source with a wavelength range overlapping the natural sunlight wavelength. As a result sixteen transformation products were identified, and 15 TPs are new compounds which structure was elucidated and reported here for the first time. It was discovered that the degradation process do not stop after the formation of the first photoproduct (amlodipine pyridine derivative), but it is progressing and reveal the existence of various compounds, some of them are created from the first photoproduct. It was also discovered that some TPs are formed in acidic (pH 3) or basic (pH 10) conditions. As a result, transformation pathway of amlodipine was proposed.

Finally, the occurrence of AML and its photoproducts was investigated in various water samples (wastewater influent and effluent, river water). Only AML 2 was detected in four wastewater effluent samples. Performing the QSAR proved that sewage sludge adsorption is not a dominant process in AML removal (except for AML 5, which sorption was estimated on level of ∼54% in STP model). In general, the values of log K_ow_ were below 4 indicating high solubility in water of most of the compounds, and the results for log K_oc_ suggested low or negligible sorption to sewage sludge and moderate migration to ground water. Further research on the presence of AML and its TPs at various stages of wastewater treatment and in ground water should be performed.

UHPLC-QTOF-MS is a very powerful tool for the identification of TPs in the aqueous environment providing accurate mass measurements, MS/MS fragmentation or isotope profiling which are of a high significance while identifying degradation products and elucidating/verifying their structure. To the best of our knowledge this is the most extensive study on amlodipine photodegradation published so far.

## Supporting Information

Figure S1 **Absorbance spectrum of amlodipine.**
(TIF)

Figure S2 **MS/MS fragmentation spectra of amlodipine and its 16 photoproducts identified by UHPLC-QTOF-MS.**
(TIF)

Table S1 **Detailed data used for photodegradation kinetics calculations (** ***initial concentration was treated as 100%*** **).**
(DOCX)
