# Supplementary figures and images for: Phototransformation of Amlodipine: Degradation Kinetics and Identification of Its Photoproducts
Source: PLoS One. 2014 Oct 3;9(10):e109206. doi: 10.1371/journal.pone.0109206 (PMC4184881; doi:10.1371/journal.pone.0109206)

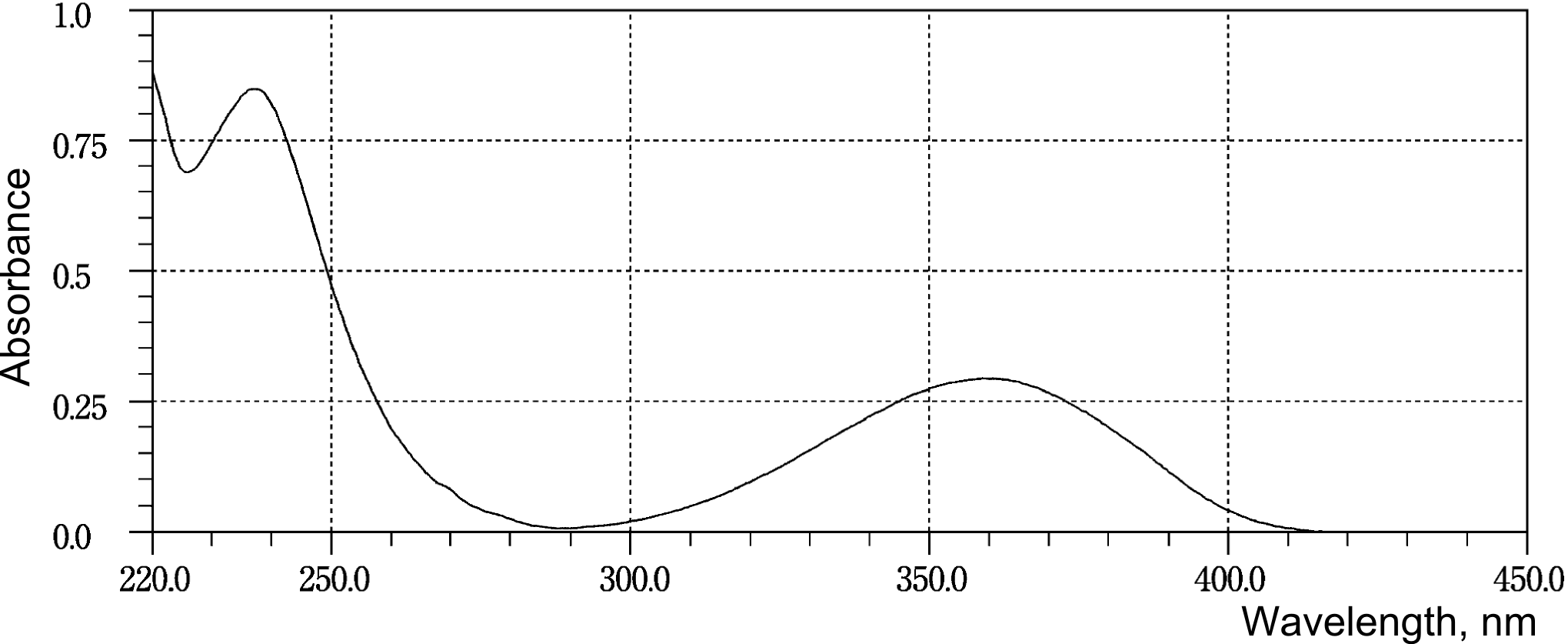

Supplement: Figure S1 — Absorbance spectrum of amlodipine. (TIF) [file pone.0109206.s001.tif]
