# Supplementary material for: Phototransformation of Amlodipine: Degradation Kinetics and Identification of Its Photoproducts
Source: PLoS One. 2014 Oct 3;9(10):e109206. doi: 10.1371/journal.pone.0109206 (PMC4184881; doi:10.1371/journal.pone.0109206)
Supplement: Table S1 — Detailed data used for photodegradation kinetics calculations ( initial concentration was treated as 100% ). (DOCX) [file pone.0109206.s003.docx]

Table S1. Detailed data used for photodegradation kinetics calculations (*initial concentration was treated as 100%*)

| Matrix | **Solar irradiation** | | | | | | | |
| --- | --- | --- | --- | --- | --- | --- | --- | --- |
|  | Irradiation time, day | Peak area | **Zero-order-kinetic** | | **First-order-kinetic** | | **Second-order-kinetic** | |
|  |  |  | **C_t_-C_0_=-kt** | **R^2^** | **ln(C_t_/C_0_)=-kt** | **R^2^** | **1/C_0_-1/C_t_=-kt** | **R^2^** |
| Wastewater influent | 0 | 87113544 | 0.00 | 0.848 | 0.00 | 0.999 | 0.00 | 0.848 |
|  | 1 | 13439320 | -84.57 |  | -1.87 |  | -0.05 |  |
|  | 2 | 2088732 | -97.60 |  | -3.73 |  | -0.41 |  |
| Wastewater effluent | 0 | 97163319 | 0.00 | 0.903 | 0.00 | 0.998 | 0.00 | 0.868 |
|  | 0.5 | 25163773 | -74.10 |  | -1.35 |  | -0.03 |  |
|  | 1 | 5243773 | -94.60 |  | -2.92 |  | -0.18 |  |
| River water | 0 | 88593558 | 0.00 | 0.914 | 0.00 | 0.998 | 0.00 | 0.883 |
|  | 0.5 | 25586382 | -71.12 |  | -1.24 |  | -0.02 |  |
|  | 1 | 6189638 | -93.01 |  | -2.66 |  | -0.13 |  |
| Untreated water | 0 | 96685007 | 0.00 | 0.863 | 0.00 | 0.990 | 0.00 | 0.801 |
|  | 1 | 16095554 | -83.35 |  | -1.79 |  | -0.05 |  |
|  | 2 | 1309698 | -98.65 |  | -4.30 |  | -0.73 |  |
| Treated water | 0 | 92340086 | 0.00 | 0.820 | 0.00 | 0.994 | 0.00 | 0.874 |
|  | 1 | 38807444 | -57.97 |  | -0.87 |  | -0.01 |  |
|  | 2 | 16260641 | -82.39 |  | -1.74 |  | -0.05 |  |
|  | 3 | 5596310 | -93.94 |  | -2.80 |  | -0.16 |  |
|  | 4 | 3059940 | -96.69 |  | -3.41 |  | -0.29 |  |
| Methanol | 0 | 101686005 | 0.00 | 0.949 | 0.00 | 0.962 | 0.00 | 0.747 |
|  | 1 | 58531134 | -42.44 |  | -0.55 |  | -0.01 |  |
|  | 2 | 14569221 | -85.67 |  | -1.94 |  | -0.06 |  |
|  | 3 | 3086488 | -96.96 |  | -3.49 |  | -0.32 |  |
| Ultrapure water pH 3 | 0 | 102530822 | 0.00 | 0.913 | 0.00 | 0.980 | 0.00 | 0.875 |
|  | 1 | 69138012 | -32.57 |  | -0.39 |  | 0.00 |  |
|  | 2 | 63667852 | -37.90 |  | -0.48 |  | -0.01 |  |
|  | 3 | 39825158 | -61.16 |  | -0.95 |  | -0.02 |  |
|  | 4 | 37499073 | -63.43 |  | -1.01 |  | -0.02 |  |
|  | 5 | 28440451 | -72.26 |  | -1.28 |  | -0.03 |  |
|  | 6 | 21581908 | -78.95 |  | -1.56 |  | -0.04 |  |
|  | 7 | 14179911 | -86.17 |  | -1.98 |  | -0.06 |  |
| Ultrapure water pH 10 | 0 | 46483171 | 0.00 | 0.863 | 0.00 | 0.994 | 0.00 | 0.889 |
|  | 1 | 17843009 | -61.61 |  | -0.96 |  | -0.02 |  |
|  | 2 | 5059703 | -89.11 |  | -2.22 |  | -0.08 |  |
|  | 3 | 2158756 | -95.36 |  | -3.07 |  | -0.21 |  |
|  | **Xenon lamp irradiation** | | | | | | | |
|  | Irradiation time, min | Peak area | **Zero-order-kinetic** | | **First-order-kinetic** | | **Second-order-kinetic** | |
|  |  |  | **C_t_-C_0_=-kt** | **R^2^** | **ln(C_t_/C_0_)=-kt** | **R^2^** | **1/C_0_-1/C_t_=-kt** | **R^2^** |
| River water | 0 | 71744468 | 0.00 | 0.738 | 0.00 | 0.999 | 0.00 | 0.729 |
|  | 15 | 21873576 | -69.51 |  | -1.19 |  | -0.02 |  |
|  | 30 | 6514968 | -90.92 |  | -2.40 |  | -0.10 |  |
|  | 45 | 1953173 | -97.28 |  | -3.60 |  | -0.36 |  |
|  | 60 | 571169 | -99.20 |  | -4.83 |  | -1.25 |  |
